# Acid-dependent beta-lactam resistance in *Klebsiella pneumoniae* is mediated by paralogous class B PBPs and the class A PBP, PBP1b

**DOI:** 10.1128/mbio.00092-26

**Published:** 2026-05-11

**Authors:** Sarah Beagle, Petra Anne Levin

**Affiliations:** 1 Department of Biology, Washington University in St. Louis 7548, St. Louis, Missouri, USA; Universite de Geneve, Geneva, Switzerland

**Keywords:** pH, antibiotic resistance, beta-lactams, pathogens, *Klebsiella*

## Abstract

*Klebsiella pneumoniae* is a leading cause of global deaths due to antibiotic resistance. Of particular concern is the rapid expansion of resistance to beta-lactam antibiotics within *K. pneumoniae* lineages. The environmental factors that influence pathogen physiology and, subsequently, antibiotic resistance remain poorly understood. Here, we demonstrate that physiologically relevant reductions in pH increased *K. pneumoniae* beta-lactam resistance as much as 64-fold, with the most dramatic increase observed for beta-lactams that specifically inhibit cell division. We identified two genes that contribute to acid-dependent beta-lactam resistance: the class A penicillin-binding protein (PBP), PBP1b, and the paralogous class B PBP, PBP3_PARA_. Loss of either PBP1b or PBP3_PARA_ increases *K. pneumoniae* susceptibility to beta-lactams at low pH. Altogether, these data emphasize the importance of functional redundancy among cell wall synthesis enzymes, which allows for specialization and ensures robust cell wall synthesis across a range of environmental conditions.

**IMPORTANCE:**

Beta-lactams are the most prescribed class of antibiotics, but their effectiveness is threatened by a global rise in antimicrobial resistance. How the environment within a host or infection site shapes pathogen response to antibiotics is frequently overlooked in assessments of antibiotic effectiveness. We demonstrate that growth at physiologically relevant low pH substantially increases *Klebsiella pneumoniae* resistance to clinically important beta-lactams. An important finding of this study is that during growth in acidic pH, *K. pneumoniae* has a different repertoire of cell wall synthesis genes available than during growth at neutral pH due to the presence of acid-inducible paralogous copies of essential cell wall synthesis enzymes, PBP2 and PBP3. An additional functionally redundant enzyme, PBP1b, also contributes to acid-dependent beta-lactam resistance. Together, these findings expand our understanding of how bacteria maintain cell wall synthesis across diverse physicochemical environments and highlight potential new therapeutic targets.

## INTRODUCTION

Infections caused by the ESKAPE pathogen *Klebsiella pneumoniae* are increasingly difficult to treat due to dramatic rises in antibiotic resistance. *K. pneumoniae* is a dominant member of two different beta-lactam-resistant groups of bacteria, extended-spectrum-beta-lactamase (ESBL)-producing *Enterobacteriaceae* and carbapenem-resistant *Enterobacteriaceae*, which are labeled serious and urgent public health threats by the CDC (1). In 2019, *K. pneumoniae* was the third leading cause of global deaths, where the death was directly due to or associated with antibiotic resistance (2). While ESBLs and related enzymes are major contributors to beta-lactam resistance in Gram-negative bacteria, data from our group and others identify physicochemical parameters consistent with a host environment as under-appreciated drivers of resistance. Changes in pH, nutrient availability, and accumulation of the alarmone, ppGpp, increase antibiotic resistance across a range of bacterial pathogens, highlighting the need to better understand how environmental factors impact pathogen physiology (3–6).

Despite the prevalence of resistance markers, such as ESBLs, beta-lactams remain the most prescribed antibiotic class (7). Beta-lactams interfere with the synthesis of the cell wall or peptidoglycan (PG), an essential structure that provides shape and structural rigidity to the bacterial cell. PG is comprised of glycan strands of repeating linked N-acetylglucosamine (NAG) and N-acetylmuramic acid (NAM) sugars, and these glycan strands are crosslinked together by short peptide chains (8). Synthesis and maintenance of PG requires many enzymatic steps that occur across multiple cellular locations; PG precursors are built in the cytoplasm, transported through the inner membrane, and fully assembled into PG within the periplasmic space. Among the *Enterobacteriaceae,* periplasmic PG assembly is highly enzymatically redundant, with an average of four enzymes capable of carrying out a single periplasmic PG synthesis reaction (9). This apparent functional redundancy is particularly striking when compared to the earlier cytoplasmic PG synthesis reactions, which occur in a nearly 1:1 enzyme-to-reaction ratio (10). While precursors are assembled in the relatively buffered cytoplasm, the cell wall itself is built in the periplasm and is exposed to the physicochemical environment.

Penicillin-binding proteins (PBPs) carry out essential transpeptidation reactions in the periplasm. Class A PBPs are bi-functional enzymes that both link the NAG-NAM sugar moieties together (transglycosylation, TG), as well as crosslink the peptide stems of adjacent glycan strands (transpeptidation, TP) (11). In *Escherichia coli,* the primary class A PBPs are PBP1a and PBP1b, which are encoded by *mrcA* and *mrcB,* respectively. PBP1a and PBP1b are individually dispensable for growth, but loss of both is lethal (12–14). A third class A PBP, PBP1c (*pbpC*), has an unknown role in PG synthesis and maintenance and is dispensable under standard laboratory conditions (15). Class B PBPs are monofunctional transpeptidases; these PBPs work in conjunction with cognate glycosyltransferases. PBP2 and PBP3 are the canonical class B PBPs in Gram-negative bacteria, and they function within the elongation and division machinery, respectively.

Substrate mimics, beta-lactams interact with residues within the PBP active site, resulting in acylation of a conserved serine residue and permanently preventing transpeptidation (7). Our previous work indicates that growth at physiologically relevant low pH (~pH 5) substantially increases *E. coli* resistance to PBP2- and PBP3-targeting beta-lactams. Both growth in pH values <5, as well as acid-dependent beta-lactam resistance, required the class A PBP, PBP1b, while full fitness during growth in alkaline pH required PBP1a (3). To determine if this response is conserved amongst other *Enterobacteriaceae*, we evaluated the impact of environmental pH on *K. pneumoniae* resistance to beta-lactams.

Here, we report that *K. pneumoniae* beta-lactam resistance increased as much as 64-fold during growth in acidic conditions. Genetic analysis identified two cell wall synthesis genes, *mrcB* and *ftsI2,* that are critical for pH-dependent changes in antibiotic resistance. *mrcB* encodes PBP1b, a bifunctional class A PBP previously identified as a contributor to pH-dependent antibiotic resistance in *E. coli* (3). Induced at low pH, *ftsI2* encodes PBP3_PARA_, a paralogous copy of the canonical, division-associated class B PBP, PBP3. PBP3_PARA_ is required for both acid-dependent beta-lactam resistance and robust growth and division at low pH. Altogether, our findings highlight that functional redundancy in bacterial PBPs confers fitness advantages and contributes to environmentally driven antibiotic resistance.

## RESULTS

### *Klebsiella pneumoniae* exhibits acid-dependent resistance to beta-lactam antibiotics

To determine the impact of pH on *K. pneumoniae* beta-lactam susceptibility, we assessed the response of a urinary tract infection isolate, *K. pneumoniae* TOP52 (16), to a panel of beta-lactams during growth across a range of physiologically relevant pH conditions. The drug panel included beta-lactams that preferentially target PBPs in the divisome (i.e., cephalexin, aztreonam, and piperacillin) or the elongasome (meropenem, doripenem), as well as non-specific inhibitors (ampicillin, carbenicillin) that are reported to have similar affinities for all PBPs (17, 18). Relative susceptibility or resistance to a given beta-lactam was determined by comparing the minimal inhibitory concentration of the drug at neutral pH to that at other pH values.

**Fig 1:**
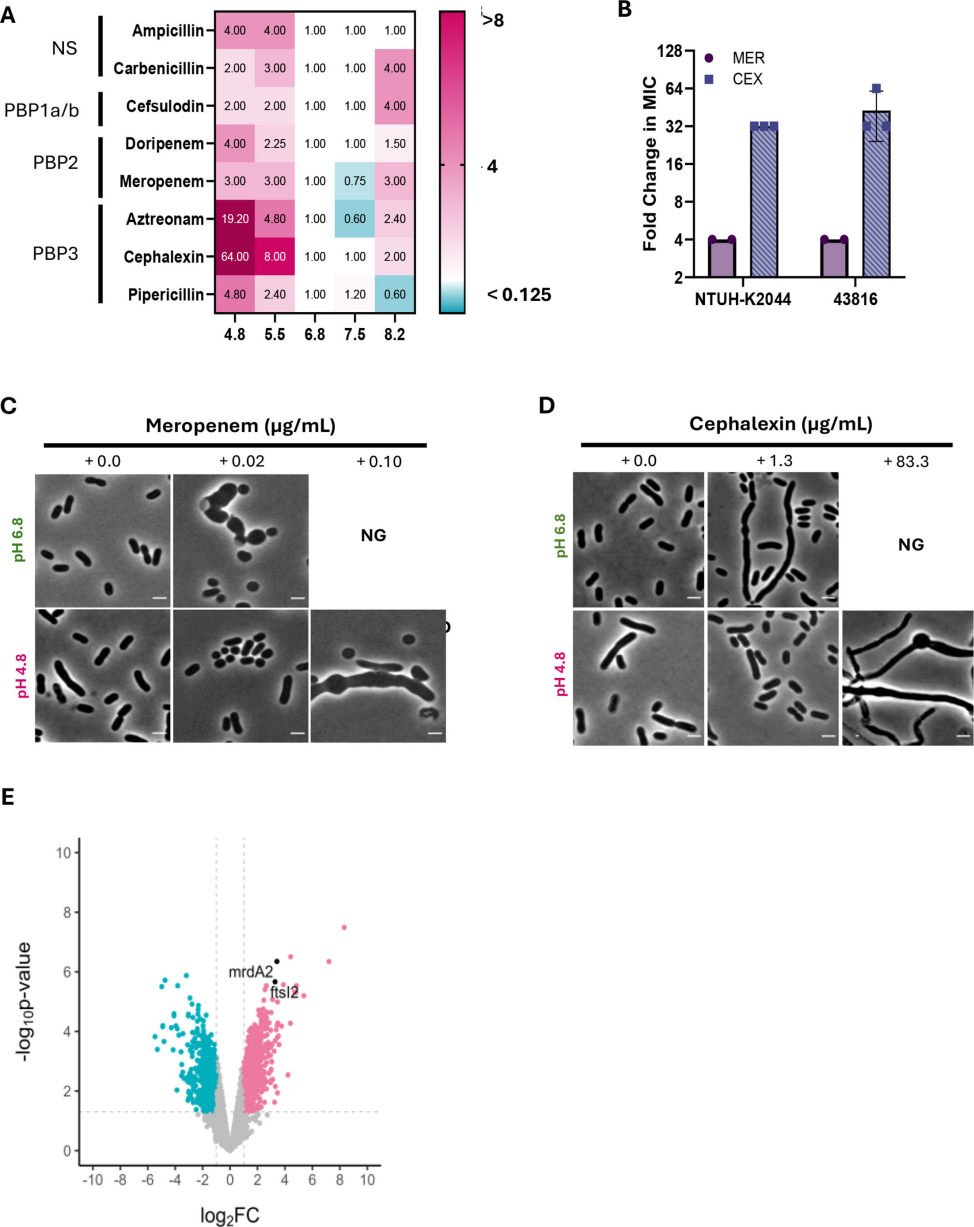
*Klebsiella pneumoniae* exhibits acid-dependent resistance to beta-lactams that is independent of beta-lactamase expression. (**A**) Heatmap displaying the fold change in minimal inhibitory change (MIC) for *K. pneumoniae* TOP52 at indicated pH relative to neutral pH. Each cell represents the median fold change value from a minimum of three biological replicates performed in LB adjusted to indicated pH with MMT buffer. Specific beta-lactam antibiotics along with their preferential PBP target are indicated on the *Y*-axis. NS = non-specific (**B**) The fold change in meropenem (MER) or cephalexin (CEX) MIC of *K. pneumoniae* strains ATCC 43816 and NHUT-K2044 during growth at pH 4.8 compared to growth at pH 6.8. Data graphed as median values with range. (**C, D**) Representative micrographs of terminal morphologies of *K. pneumoniae* TOP52 following growth for 20 hours in LB (pH 4.8 or 6.8) in the presence of meropenem (**C**) or cephalexin (**D**) at indicated concentrations. Scale bar = 2 µm. NG = no growth. (**E**) Summary of RNA sequencing results comparing gene expression of acid-cultured cells to that of cells grown under neutral pH. Genes that are downregulated during growth at low pH are designated in blue, genes that are not differentially regulated are shown in gray, and genes that are upregulated during growth at low pH are represented in magenta. The two black data points represent the expression data for the class B PBP paralogous genes.

*K. pneumoniae* exhibited a 2- to 64-fold increase in resistance to the entire spectrum of tested beta-lactams when grown in LB at pH values <5 relative to resistance at pH 7 (Fig. 1; Table S1). We observed strong acid-dependent increases in resistance for drugs that preferentially target PBP3, with aztreonam (AZT) and cephalexin (CEX) minimal inhibitory concentrations (MICs) increasing as much as 20- and 64-fold, respectively, at pH 4.8. Resistance to PBP2-targeting beta-lactams increased modestly at low pH, with MICs to doripenem and meropenem (MER) increasing 2- to 4-fold. In contrast to *E. coli* (3), we also observed a modest 2- to 4-fold increase in resistance to the non-specific beta-lactams, ampicillin and carbenicillin, at low pH. At these pH values, increases in MICs during growth at low pH are not due to significant antibiotic degradation (3).

Low pH not only enabled growth at concentrations of drug that are lethal at neutral pH but also helped preserve cell morphology. Consistent with increased intrinsic resistance at low pH*, K. pneumoniae* better maintained its characteristic rod-shaped morphology during growth under acidic conditions in concentrations of beta-lactams that cause either cell rounding (meropenem) or filamentation (cephalexin) at neutral pH (Fig. 1C and D; Fig. S1). The observed acid-dependent increase in beta-lactam resistance is not strain-specific behavior, as *K. pneumoniae* strains NTUH-K2044 and ATCC 43816 also exhibited a fourfold increase to MER and an ~32-fold increase to CEX when grown at pH 4.8 versus pH 6.8 (Fig. 1B).

To determine if this behavior also occurs under conditions that better reflect a native host environment, we assayed how *K. pneumoniae* responded to beta-lactams during growth in an artificial urine growth medium at low and neutral pH (19). Similar to growth in LB, we observed 32- to >64-fold increases in resistance to ampicillin and cephalexin in acidified artificial urine with and without amino acid supplementation compared to neutral pH (Fig. S2, Tables S1 and S2). While the MIC for a given beta-lactam was generally higher during growth at low pH, independent of media composition, we observed that for certain antibiotics, the media composition, coupled with acidification of the media, significantly altered the susceptibility. The MIC for ampicillin at low pH in LB was over 20-fold lower (mean MIC = 104 µg/mL ± 37) than during growth in AU (mean MIC = 2,500 µg/mL ± 0.0), indicating an interplay between nutrient composition and pH. We were unable to determine the exact fold change in MIC for cephalexin, as *K. pneumoniae* grew in all tested concentrations of cephalexin in both AU and AU+AA (Fig. S2; Table S2). Together, our data suggest that acid-dependent beta-lactam resistance occurs across a range of nutrient conditions and is conserved amongst *K. pneumoniae* lineages.

### pH modulates expression of class B PBP paralogs in *K. pneumoniae*

To ascertain the impact of pH on the expression of genes encoding PBPs or other cell wall synthesis genes, we performed RNAseq in *K. pneumoniae* comparing cells cultured in LB at pH 4.8 versus pH 6.8, hereafter referred to as low and neutral pH (Fig. 1E; Table S3). Consistent with previous studies, pH does not impact expression of class A PBPs, *mrcA* (PBP1a) or *mrcB* (PBP1b), in either *E. coli* or *K. pneumoniae* (20, 21) (Table 1)*.* Class A PBP activity is regulated by cognate outer membrane activators, LpoA and LpoB (22–25). We observed that *lpoB,* which encodes the PBP1b activator, was down-regulated approximately twofold at low pH. PBP1c, a cryptic class A PBP with no demonstrated role in cell wall synthesis under standard laboratory conditions, exhibited an approximately twofold decrease in expression during growth in acidic pH (Table 1; Table S3). We observed no significant changes in class B PBP expression between pH conditions, but the carboxypeptidases PBP5 (*dacA*) and PBP6b (*dacD*) exhibited a modest twofold to threefold increase in expression during growth at low pH. Both *dacA* and *dacD* are implicated in maintaining proper cell morphology in *E. coli*, with *dacD* being required for proper morphology specifically at low pH (26, 27).

**TABLE 1:** Transcriptional response to pH of key beta-lactam targets in *K. pneumoniae* and *E. coli^a^ *

	Gene	Enzyme encoded	Description	Log_2_FC (pH 4.8/6.8)
*K. pneumoniae*				
D1637_RS05825	*mrcA*	PBP1a	Bifunctional glycosyl transferase/transpeptidase	0.60
D1637_RS12385	*mrcB*	PBP1b	Bifunctional glycosyl transferase/transpeptidase	−0.39
D1637_RS01040	*pbpC*	PBP1c	Peptidoglycan glycosyltransferase	**−1.61**
D1637_RS15400	*mrdA*	PBP2	Canonical peptidoglycan DD-transpeptidase	0.84
D1637_RS12000	*ftsI*	PBP3	Canonical peptidoglycan DD-transpeptidase	0.23
D1637_RS21590	*mrdA2*	PBP2_para_	Paralogous peptidoglycan DD-transpeptidase	*3.41*
D1637_RS24275	*ftsI2*	PBP3_para_	Paralogous peptidoglycan DD-transpeptidase	*3.28*
D1637_RS15385	*dacA*	PBP5	D-Ala-D-Ala-carboxypeptidase	*1.20*
D1637_RS25010	*dacD*	PBP6b	D-Ala-D-Ala carboxypeptidase	*1.63*
D1637_RS17685	*lpoB*	LpoB	Outer membrane activator of PBP1b	**−1.12**
*E. coli*				
b3396	*mrcA*	PBP1a	Bifunctional glycosyl transferase/transpeptidase	0.36
b0149	*mrcB*	PBP1b	Bifunctional glycosyl transferase/transpeptidase	−0.23
b2519	*pbpC*	PBP1c	Peptidoglycan glycosyltransferase	−0.43
b0635	*mrdA*	PBP2	Canonical peptidoglycan DD-transpeptidase	0.47
b0084	*ftsI*	PBP3	Canonical peptidoglycan DD-transpeptidase	−0.64
b0632	*dacA*	PBP5	D-alanyl-D-alanine carboxypeptidase	0.95
b2010	*dacD*	PBP6b	D-alanyl-D-alanine carboxypeptidase	*2.63*
B1105	*lpoB*	LpoB	Outer membrane activator of PBP1b	**−1.33**

^*a*^ Shaded genes represent those that were differentially regulated by pH as determined by our cutoff of |logFC| >1 and *P* <0.05. Bold log_2_FC values indicate that the gene was downregulated during growth at pH 4.8 versus pH 6.8. Italic log_2_FC values indicate that the gene was upregulated during growth at pH 4.8 versus pH 6.8.

**Fig 2:**
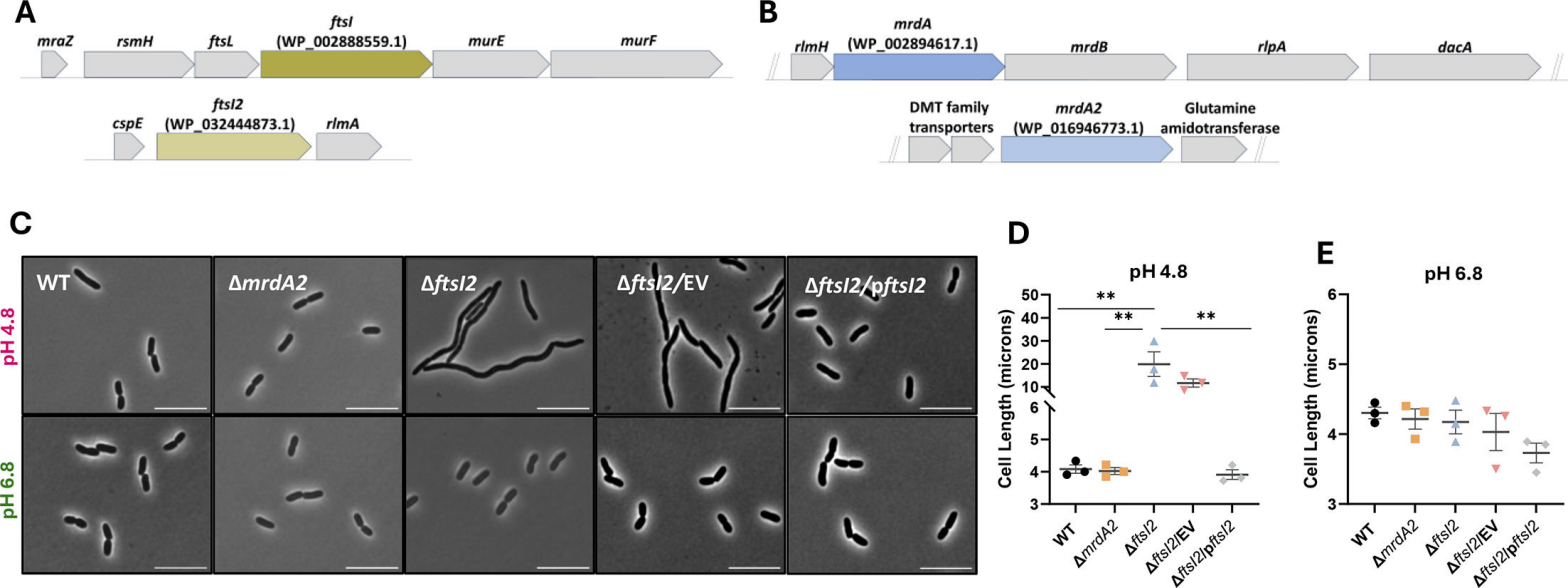
Loss of the acid-responsive PBP3_PARA_ results impairs division in *K. pneumoniae* at low pH. (**A**) Genomic regions containing the canonical PBP3, encoded by *ftsI,* and the paralogous gene *ftsI2,* which encodes PBP3_PARA_. (**B**) Genomic regions containing the canonical PBP2, encoded by *mrdA*, and the paralogous gene *mrdA2,* which encodes PBP2_PARA_. (**C**) Representative micrographs of indicated strains following growth in LB adjusted to pH 4.8 with MMT buffer (top panels) or growth in LB adjusted to pH 6.8 with MMT buffer (bottom panels). Scale bar = 10 µm. EV = empty vector and p*ftsI2 =* IPTG-inducible copy of *ftsI2.* (**D and E**) Quantification of cell length in panel **C**. Each data point represents the mean cell length of a biological replicate (>50 cells analyzed for each replicate). The bar represents the mean of three biological replicates, and the error bars represent the standard error of the mean. Statistical significance was determined by a one-way ANOVA with Tukey’s correction for multiple comparisons with asterisks denoting significance as follows: ** = *P* < 0.005.

Duplications of the class B PBPs, PBP2 and PBP3, that have expanded the repertoire of cell wall synthesis enzymes have been described in several pathogens (28–30). Unexpectedly, our RNA sequencing data revealed that *K. pneumoniae* also encodes paralogous copies of both class B PBPs. We designated the paralogous genes as *mrdA2* and *ftsI2,* encoding the transpeptidases PBP2_PARA_ and PBP3_PARA_, respectively (Fig. 1E and 2A and B). Expression of *mrdA2* and *ftsI2* increased by ~10-fold during growth at low pH, making them the most upregulated PG synthesis genes at low pH.  

### Loss of PBP3_PARA_ results in filamentation in *K. pneumoniae* at low pH

Given the dramatic increase in expression at low pH, we next wanted to explore the role that the paralogous class B PBPs play in cell wall synthesis, shape maintenance, and division in *K. pneumoniae.* In *Salmonella enterica* serovar Typhimurium (*S.* Typhimurium), the class B PBP paralogs, PBP2_SAL_ and PBP3_SAL_, are necessary for optimal fitness during growth at low pH. Expression of both PBP2_SAL_ and PBP3_SAL_ is maximally induced at low pH (<5.8), and PBP2_SAL_ helps maintain rod-shaped morphology under acidic environments (29, 30). Like their *Salmonella* counterparts, PBP2_PARA_ and PBP3_PARA_ in *Klebsiella* share significant sequence identity with the canonical class B PBPs (59% for PBP2_PARA_ and 61% for PBP3_PARA_) and retain the sequence motifs associated with catalysis (Fig. S3).

To evaluate the role of class B PBP paralogs in *K. pneumoniae* cell morphology and division, we generated individual gene deletion strains and examined mutants at early exponential phase (OD_600_ = 0.1–0.2) using cell size analysis and time-lapse microscopy under various pH conditions. While loss of PBP2_PARA_ (Δ*mrdA2*) did not impact *K. pneumoniae* cell size or morphology at either pH, we found that during growth in acidified medium, loss of PBP3_PARA_ (Δ*ftsI2*) resulted in heterogeneous cell lengths, with many cells displaying filamentous behavior indicative of cell division defects (Fig. 2C). Using a definition of filamentation as a cell length of >15 µm which represents ~3× the average length of WT cells at pH 4.8 (3.9 ± 0.2 µm; see Fig. 4B), we determined that 21%–58% of Δ*ftsI2* cells within a given population were filamentous, with a smaller proportion of cells (3%–17%) reaching lengths of >40 µm (Fig. S4).

The division defects associated with loss of PBP3_PARA_ were specific to growth in acidic conditions. Time-lapse microscopy of acid-grown Δ*ftsI2* cells spotted onto acidic LB agarose pads revealed a population of cells with heterogeneous lengths as cells continued to elongate without successful division, resulting in long filamentous cells. In contrast, spotting acid-grown Δ*ftsI2* cells onto LB agarose pads at neutral pH restored division to the filamentous cells (Fig. S5). Additionally, growth of the Δ*ftsI2* mutant at neutral pH resulted in an average cell length consistent with WT cell lengths (Fig. 2E). Complementation of *ftsI2* in *trans* from a medium-copy number plasmid enabled division and restored cell length to values consistent with WT at low pH (Fig. 2D). We attempted to disrupt the canonical PBP3 under acidic culture conditions where PBP3_PARA_ is likely the predominant division-specific transpeptidase; however, despite multiple attempts, we were unsuccessful. Moreover, a small percentage of Δ*ftsI2* cells maintained WT lengths at low pH, suggesting that the canonical PBP3 retains limited, but critical activity at low pH (Fig. S4). These data differ from *Salmonella,* where the canonical PBP3 and the paralog PBP3_SAL_ are individually dispensable at low pH, but PBP3_SAL_ is unable to support division at neutral pH (29). Together, these data suggest that PBP3_PARA_ is the predominant transpeptidase supporting cell division in *K. pneumoniae* at low pH.

### Genetic data suggest pH-dependent *ftsI2* expression requires the transcriptional regulator OmpR

**Fig 3:**
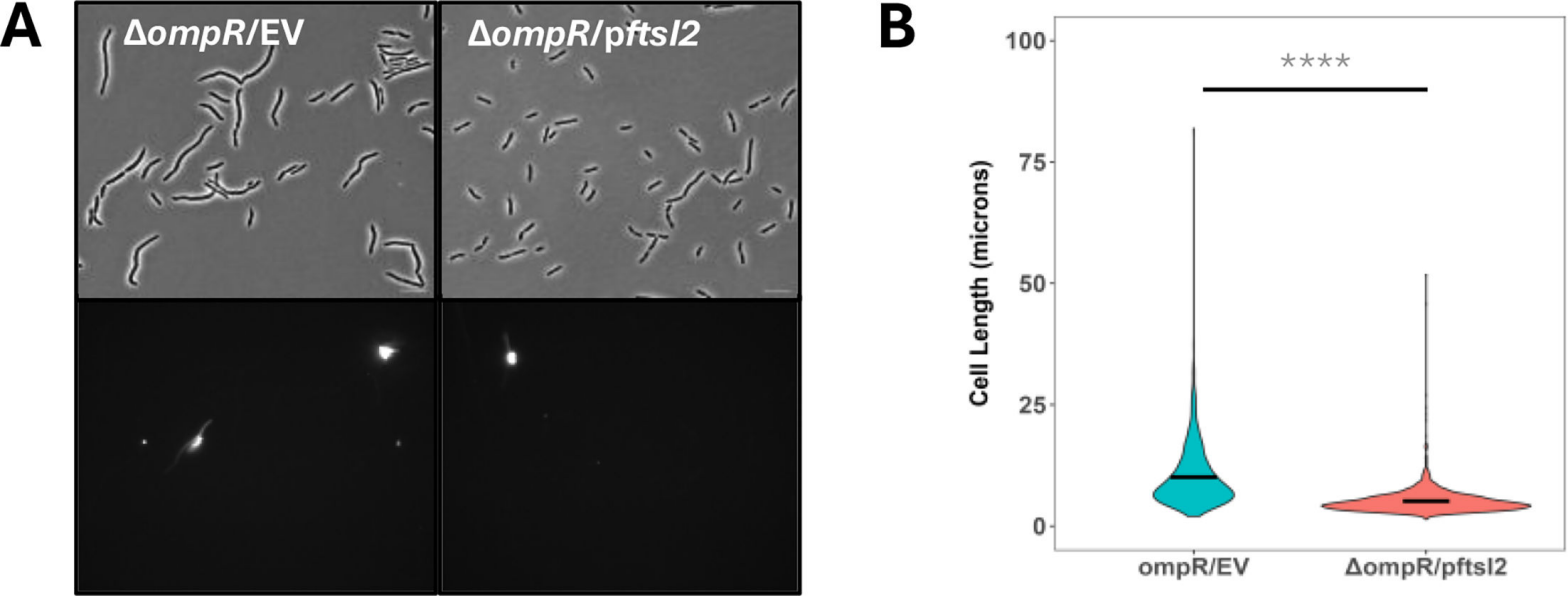
Exogenous expression of *ftsI2* improves division in acidic pH in a Δ*ompR* mutant. (**A**) Representative micrographs of a *K. pneumoniae* Δ*ompR* mutant carrying either an empty vector (EV) or an IPTG-inducible copy of *ftsI2* (p*ftsI2*). Microscopy pads are comprised of LB adjusted to pH 4.8 with MMT buffer, 1% agarose, and 1.5 µM propidium iodide to monitor membrane integrity. Top panels represent phase contrast images, and bottom panels are the corresponding fluorescent images. Scale bar = 10 µm. (**B**) Quantification of cell lengths from phase contrast images in panel **A**. Statistical significance was determined by a Mann-Whitney *U* test with asterisks denoting significance as follows: **** = *P* < 0.0001.

The acid-responsive expression of the paralogous *K. pneumoniae* class B PBPs suggests alternate transcriptional regulation as compared to their canonical counterparts. In *S.* Typhimurium, the paralogous class B PBPs are under the regulation of EnvZ/OmpR, a two-component system involved in response to osmotic and acid stress (31–33). We generated a response regulator deletion mutant (Δ*ompR*) and assayed how the strain behaved during growth at low and neutral pH. We reasoned that if OmpR activates *ftsI2* expression in response to acidic pH, then loss of the response regulator should lead to filamentation at low pH. Consistent with that hypothesis, the Δ*ompR* mutant exhibited filamentation and increased lysis during growth at low pH (Fig. 3A). Expression of an IPTG-inducible copy of *ftsI2* (p*ftsI2*) restored division in the Δ*ompR* mutant at low pH, with the Δ*ompR*/*pftsI2* strain averaging a cell length of 5.2 ± 2.8 µm as compared to an average length of 10.1 ± 6.5 µm for the Δ*ompR* strain carrying an empty vector (Fig. 3B). Our data indicates that the division defects observed in the Δ*ompR* strain during growth at low pH are due to impaired expression of *ftsI2.*

### Investigating the genetic determinants underlying acid-dependent beta-lactam resistance

To gain a better understanding of the genetic requirements underlying acid-dependent beta-lactam resistance, we assayed the response of a targeted panel of mutants to beta-lactam antibiotics during growth at low and neutral pH. We focused on cell wall synthesis genes that were acid-responsive in our RNAseq data set, along with those that have been implicated in PG synthesis, shape maintenance, or antibiotic resistance in *E. coli* and other bacteria.

**Fig 4:**
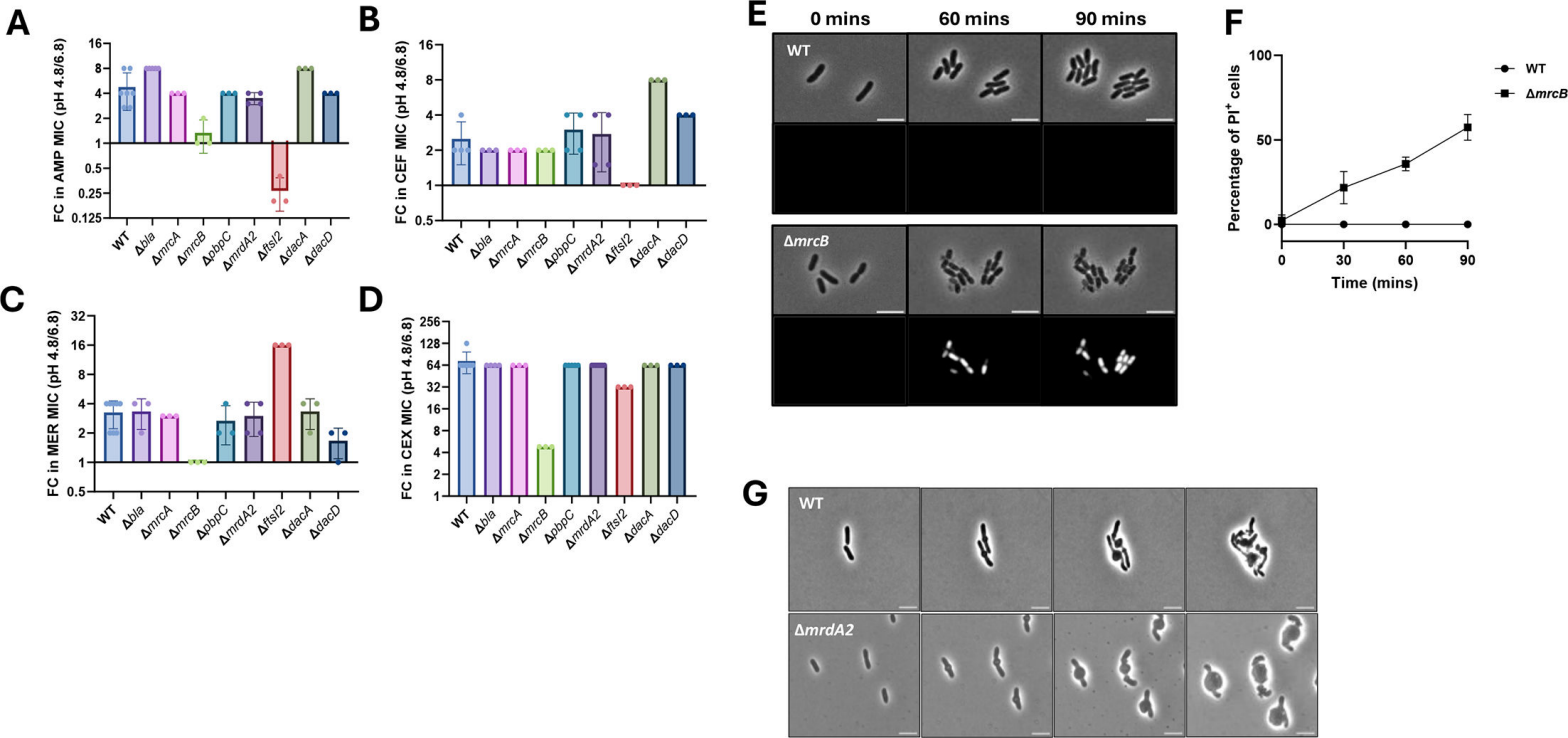
Loss of either PBP1b or PBP3_PARA_ results in differential impacts on beta-lactam resistance at low pH. (**A–D**) Fold change in MICs at pH 4.8 relative to pH 6.8 is shown for generalist (**A**), class A-targeting (**B**), PBP2-targeting (**C**), and PBP3-targeting (**D**) beta-lactams. Data are graphed as median values with range. (**E**) Micrographs from time lapse microscopy of WT (top) or Δ*mrcB* (bottom) growing in the presence of CEX at low pH. Microscopy pads comprised of LB buffered with MMT to pH 4.8 + 15.6 µg/mL CEX + 1.5 µM propidium iodide + 1% agarose. The CEX concentration reflects the 1× MIC for the Δ*mrcB* mutant and 0.06× MIC for the WT strain. (**F**) Quantification of the percentage of cells staining with propidium iodide (a marker for loss of membrane integrity and viability) within the first 90 min of CEX exposure. (**G**) Micrographs from time lapse microscopy of WT and Δ*mrdA2* growing in the presence of MER at low pH. Microscopy pads comprised of LB buffered with MMT to pH 4.8 + 0.2 µg/mL MER + 1.5 µM propidium iodide + 1% agarose. The MER concentration reflects 1× MIC for WT and *~*1.3× MIC for Δ*mrdA2*. CEX, cephalexin; MER, meropenem.

Of the mutants we examined, only those defective for the genes encoding the functionally redundant PBPs, PBP1b and PBP3_PARA_, were impaired in pH-mediated resistance (Fig. 4A through D; Table S4). Importantly, while loss of TOP52’s endogenous class-A beta-lactamase, *bla_SHV-1_*, reduced resistance to specifically the Penicillin sub-class of beta-lactams as expected (7, 16, 34), ∆*bla_SHV-1_* mutants still exhibit fold changes in resistance on par with the wild-type parental strain (Fig. 4A through D; Table S4). This finding demonstrates that acid-dependent resistance is independent of the endogenous beta-lactamase.

### The class A PBP, PBP1b, is important for acid-dependent beta-lactam resistance in *K. pneumoniae*

*K. pneumoniae* encodes three class A PBPs, but only the loss of PBP1Bb (Δ*mrcB*) impaired *K. pneumoniae’s* resistance to beta-lactams at low pH. The Δ*mrcB* mutant eliminated the acid-dependent resistance to ampicillin, doripenem, and meropenem, and impaired resistance to cephalexin at low pH (Fig. 4; Table S4). Interestingly, a *K. pneumoniae* Δ*mrcB* strain was able to grow at low pH (Fig. S6); whereas, in *E. coli, mrcB* is required for growth under the same conditions (3).

Loss of PBP1b (Δ*mrcB*) most strongly impacted acid-mediated cephalexin resistance. Growth at low pH only increased cephalexin resistance fourfold in a Δ*mrcB* mutant compared to a 64- to 128-fold increase in MIC observed in the WT strain. While filamentation is a characteristic morphology associated with division-targeting beta-lactams (35, 36), at low pH, a PBP1b-deficient *K. pneumoniae* strain is sensitive to cephalexin at concentrations that do not elicit filamentation or lysis in WT (Fig. 4E and F). The death of the PBP1b mutant is rapid, with more than 50% of the population exhibiting lysis or loss of membrane integrity as denoted by propidium iodide staining within 90 min of exposure to cephalexin at low pH (Fig. 4F).

The ∆*mrcB* mutant also no longer exhibited acid-dependent resistance to the generalist, ampicillin, or to PBP2-targeting beta-lactams, doripenem and meropenem (Fig. 4A and C; Fig. S7, Table S4). As we observed in *E. coli* (3), PBP1b-mediated beta-lactam resistance required its cognate outer membrane activator, lpoB (Δ*lpoB*) (Fig. S7, Table S4). Defects in PBP1b also led to a nearly 10-fold increase in susceptibility to cefsulodin in both acidic and neutral pH compared to WT (Table S4). This pH-independent increase in sensitivity of the Δ*mrcB* mutant is consistent with data suggesting that PBP1a is the primary class A target of cefsulodin (37, 38).

### Loss of PBP3_PARA_, but not PBP2_PARA_, impacts beta-lactam resistance in *K. pneumoniae*

**Fig 5:**
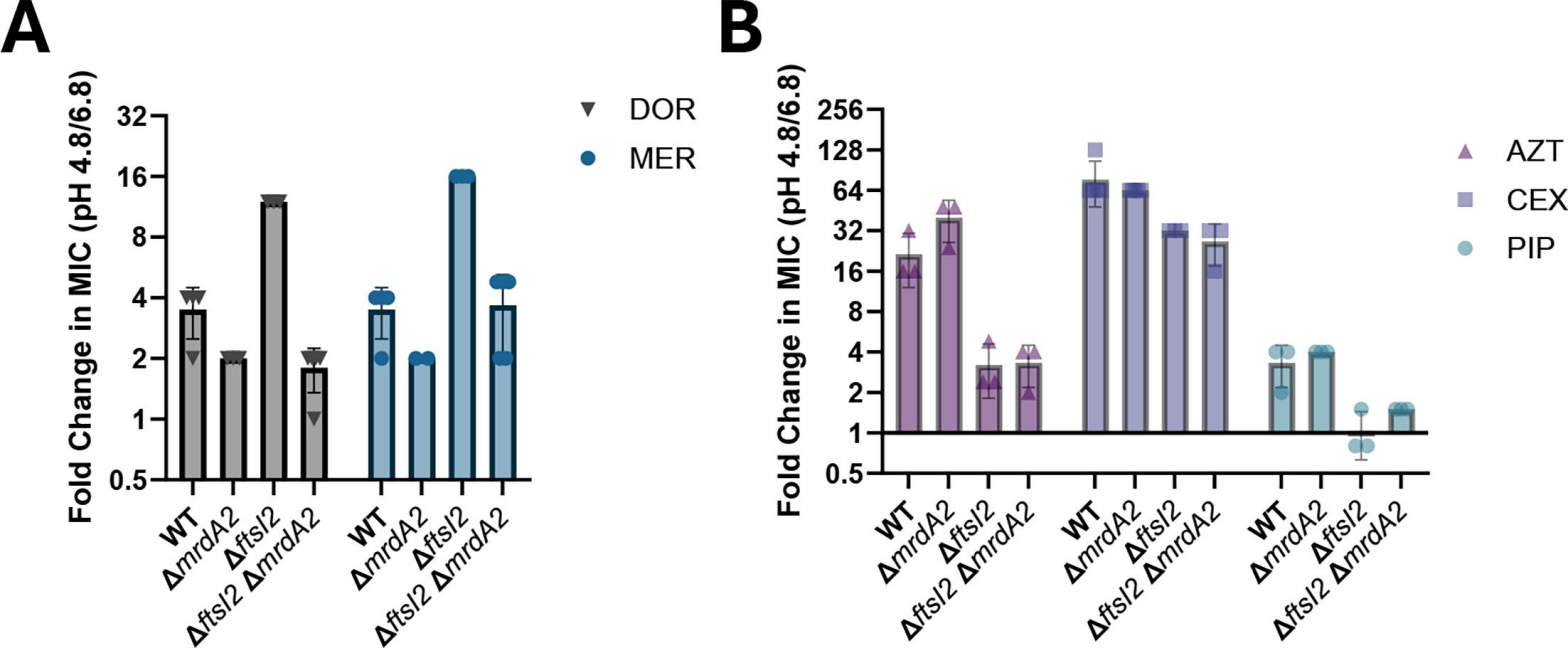
Loss of PBP3_PARA_ leads to increased resistance to PBP2-inhibitors and increased sensitivity to PBP3-inhibitors. (**A and B**) Fold change in MICs at pH 4.8 relative to pH 6.8 is shown for PBP2-targeting (**A**) and PBP3-targeting (**B**) beta-lactams. Data are graphed as median values with range. For comparison, data for MER and CEX MICs for WT and single mutants are re-graphed from Fig. 2. CEX, cephalexin; MER, meropenem.

The upregulation at low pH of *ftsI2* and *mrdA2,* encoding PBP3_PARA_ and PBP2_PARA_, respectively, suggested that the paralogous transpeptidases might play a role in the acid-dependent beta-lactam resistance phenotypes we observe in *K. pneumoniae.* Consistent with this idea, loss of *ftsI2,* encoding a paralogous copy of the divisome-specific transpeptidase, differentially impacted beta-lactam resistance at low pH. The Δ*ftsI2* mutant no longer displayed acid-dependent resistance to cefsulodin or ampicillin (Fig. 4A and B), with the Δ*ftsI2* mutant being more sensitive to ampicillin at low pH (Fig. 4A). When challenged with PBP3-targeting beta-lactams (CEX, AZT, PIP), the PBP3_PARA_-null mutant still retained mild acid-dependent resistance but was twofold to eightfold more sensitive at low pH than WT (Fig. 4D and 5B). Supplementation with p*ftsI2* restored WT levels of resistance to ampicillin and the PBP3-specific beta-lactam, piperacillin, at low pH (Fig. S8). Induction of p*ftsI2* did not alter resistance to the PBP3-targeting beta-lactam, cephalexin, for either WT or a Δ*ftsI2* mutant during growth at neutral pH (Fig. S9). In contrast to *ftsI2,* loss of *mrdA2* did not significantly alter resistance to any tested beta-lactams (Fig. 4A through D and 5). This contrasts with the increased resistance of *K. pneumoniae* to PBP2 inhibitors at low pH. It is, however, consistent with our observation that *mrdA2* mutants retain their rod shape morphology at neutral and low pH (Fig. 2C). While wild-type cells exhibited a hybrid morphology at low pH when challenged with Meropenem, initially elongating, followed by septal bulging and further elongation, the Δ*mrdA2* mutant phenotype exhibited far less elongation, becoming nearly spherical at midcell (Fig. 4G). This suggests that while its loss does not dramatically impair either drug resistance or morphology at low pH, PBP2_PARA_ may supplement the activity of its canonical partner, PBP2, with regard to elongasome activity.

### PBP2_PARA_ is required for increased acid-dependent resistance to PBP2-targeting compounds in the PBP3_PARA_ null mutant

Unexpectedly, the PBP3_PARA_ null mutant exhibited increased resistance when challenged with PBP2-targeting beta-lactams at low pH. The meropenem and doripenem MICs obtained for the Δ*ftsI2* mutant were 12–16-fold higher at acidic pH versus neutral pH. For comparison, WT cells only exhibited a 2- to 4-fold increase in MIC to these two drugs at acidic pH (Fig. 4C and 5A). We reasoned that the unexpected increased resistance to PBP2-inhibitors at low pH observed in the Δ*ftsI2* mutant could be influenced by the presence of PBP2_PARA_. To address this possibility, we generated a double deletion strain lacking both class B paralogs (Δ*ftsI2* Δ*mrdA2*) and assayed its response to beta-lactams with high specificity for PBP2 or PBP3 across pH conditions. Deletion of both class B PBP paralogs eliminated the dramatic increase in resistance at low pH, restoring WT-level responses to both doripenem and meropenem. When challenged with PBP3-targeting beta-lactams, the Δ*ftsI2* Δ*mrdA2* double mutant phenocopied the Δ*ftsI2* mutant, displaying similar reductions in resistance at low pH (Fig. 5B). Together, these data suggest that both PBP2_PARA_ and PBP3_PARA_ are involved in mediating resistance to PBP2-inhibitors, whereas resistance to PBP3-targeting beta-lactams is primarily facilitated by PBP3_PARA_.

## DISCUSSION

### Acid-dependent beta-lactam resistance in *K. pneumoniae* differs mechanistically from *E. coli*

As observed in *E. coli,* beta-lactam resistance in *K. pneumoniae* increases substantially during growth in acidified medium (Fig. 1A). Importantly, we observed pH-dependent increases in beta-lactam resistance in LB, as well as in artificial urine, which mimics more physiologically relevant conditions (Fig. 1; Fig. S2). While both *E. coli* and *K. pneumoniae* demonstrate acid-dependent beta-lactam resistance, factors influencing this phenomenon differ between the two organisms. Acid-mediated beta-lactam resistance in *E. coli* appears to be solely due to intrinsic properties of the PBP enzymes, particularly PBP1b^3^, while in *K. pneumoniae,* the contribution of PBP1b is supplemented by pH-dependent induction of genes encoding paralogous class B PBPs. We find that PBP1B and the division-specific paralog, PBP3_PARA_, are major genetic contributors to acid-mediated beta-lactam resistance in *K. pneumoniae.*

Disruption of *mrcB*, the gene encoding PBP1b, eliminates acid-mediated resistance to the PBP2 inhibitors, meropenem and doripenem, and significantly reduces resistance to the PBP3-targeting drug, cephalexin. Deletion of *ftsI2,* encoding the division-specific paralogous transpeptidase PBP3_PARA_, differentially impacts beta-lactam resistance at low pH. A Δ*ftsI2* mutant displays sensitization toward ampicillin, cefsulodin, and, to different degrees, all PBP3-targeting beta-lactams, but exhibits increased resistance to PBP2-targeting antibiotics (Fig. 4A through D and 5). Notably, loss of PBP1b or its cognate outer membrane activator, LpoB, or PBP3_PARA_ did not significantly alter MICs across beta-lactam classes at neutral pH (Table S4), indicating that PBP1b and PBP3_PARA_ specifically mediate beta-lactam resistance at low pH in *K. pneumoniae*.

### PBP1b is a bifunctional class A PBP required for beta-lactam resistance at low pH

*K. pneumoniae,* like most *Enterobacteriaceae* members, encodes three class A PBP enzymes: PBP1a, PBP1b, and PBP1c. Class A PBPs have historically been described as functionally redundant, with the cell requiring either PBP1a or PBP1b for viability (12). However, recent work has highlighted the possibility of specialized roles for the class A PBPs. In *E. coli,* PBP1b is required for the *de novo* synthesis of PG (39), beta-lactam resistance (3, 40), and response to osmotic stress (41). Our data in *K. pneumoniae* also support a role for PBP1b in beta-lactam resistance*.* We observed impairment of PBP2- and PBP3-targeting-beta-lactam resistance in a Δ*mrcB* mutant at low pH but minimal impact on resistance at neutral pH. This result suggests that PBP1a compensates for the loss of PBP1b at neutral pH but is unable to fully do so under acidic environments, consistent with prior work indicating that PBP1a does not function optimally in low pH conditions (3).

These data are consistent with our previous work in *E. coli,* indicating that the activity of PBP1b is critical for response to beta-lactam stress (3). The mechanism(s) underlying PBP1b’s contribution to acid-dependent beta-lactam resistance is unclear. PBP1b could compensate for reductions in PBP2 and PBP3 activity that render cells resistant to class B-targeting beta-lactams. PBP1b compensation could be direct via its own intrinsic enzymatic activity or indirectly mediated by interactions with other division and elongation proteins (42–44). In *K. pneumoniae,* PBP1b may also facilitate pH-mediated resistance by facilitating recruitment of the acid-responsive class B PBP paralogs, PBP2_PARA_ and PBP3_PARA_, to the elongasome and divisome, respectively. This function would be consistent with our data, indicating that both PBP1b and PBP3_PARA_ are critical for pH-mediated resistance to PBP2- and PBP3-targeting beta-lactams.

### Class B PBP paralogs differentially modulate pH-dependent changes in beta-lactam resistance

Growth in acidic environments induces the expression of paralogous class B PBPs in *K. pneumoniae*. Loss of the elongation-specific transpeptidase paralog, PBP2_PARA_, did not strongly impact beta-lactam resistance at low pH, nor did it result in morphological defects during growth in acidic environments. However, the Δ*mrdA2* mutant did exhibit increased cell rounding compared to WT during growth at low pH in the presence of the PBP2 inhibitor, meropenem, suggesting that *mrdA2* does support Elongasome function under stress conditions.

Deletion of *ftsI2,* which encodes the division-specific paralogous transpeptidase PBP3_PARA_, strongly impacted antibiotic resistance during growth at low pH. The Δ*ftsI2* mutant exhibited impaired resistance to PBP3-targeting antibiotics and was sensitized to the generalist, ampicillin, during growth in acidified medium. Loss of *ftsI2* did not strongly impact antibiotic resistance or cell length at neutral pH, and induction of p*ftsI2* in a WT background did not increase resistance to divisome-targeting beta-lactams during growth at neutral pH. Together with our data indicating that PBP3_PARA_ is required for efficient division during *K. pneumoniae* growth at low pH, we speculate that PBP3_PARA_ assumes the role of the primary division-specific transpeptidase during growth in acidic environments. It is possible that PBP3_PARA_ exhibits lower affinity for certain beta-lactams and supports division under conditions where the canonical PBP3 has reduced activity, contributing to the increased resistance observed at low pH.

Unexpectedly, the Δ*ftsI2* mutant exhibited increased resistance at low pH to the PBP2-targeting beta-lactams, doripenem and meropenem. Increased PBP2 inhibitor resistance was dependent on *mrdA2,* which encodes the paralogous elongation-specific transpeptidase PBP2_PARA_. Loss of both paralogs restored WT levels of acid-dependent resistance to PBP2-targeting beta-lactams. It is unclear why the loss of PBP3_PARA_ renders cells resistant to PBP2-targeting compounds. In *E. coli,* the elongasome and divisome compete for the cell wall precursor, lipid II. Thus, one possibility is that loss of Δ*ftsI2* and the subsequent impairment of divisome function increases elongasome activity, resulting in more PG substrate to compete with the beta-lactam for access to the PBP2 binding site, increasing the MIC. This model is consistent with the observation that loss of both PBP2_PARA_ and PBP3_PARA_ restores wild-type levels of acid-dependent beta-lactam resistance (Fig. 5). However, we note that loss of PBP2_PARA_ neither increases resistance to PBP3-targeting beta-lactams nor impairs resistance to PBP2-targeting antibiotics. Additional work is needed to fully understand the complex role that the class B PBP paralogs play in resistance to PBP2-targeting beta-lactams.

### Expansion and pH specialization of cell wall synthesis enzymes provide novel therapeutic targets for pathogens

Colonization of a host exposes a bacterial pathogen to a wide range of pH conditions (45–47). We examined how changing a single growth parameter, environmental pH, altered growth and antibiotic resistance in the human pathogen, *K. pneumoniae*. In this study, we demonstrate that the acidification of growth medium to physiologically relevant levels is sufficient to increase resistance to beta-lactams, one of our most critical and widely used classes of antibiotics. We identified two functionally redundant cell synthesis enzymes, PBP1b and PBP3_PARA_, that are critical for PG maintenance and beta-lactam resistance during growth at low pH. PBP1b is one of three encoded bifunctional class A PBPs, and PBP3_PARA_ is a division-specific paralogous class B PBP.

**Fig 6:**
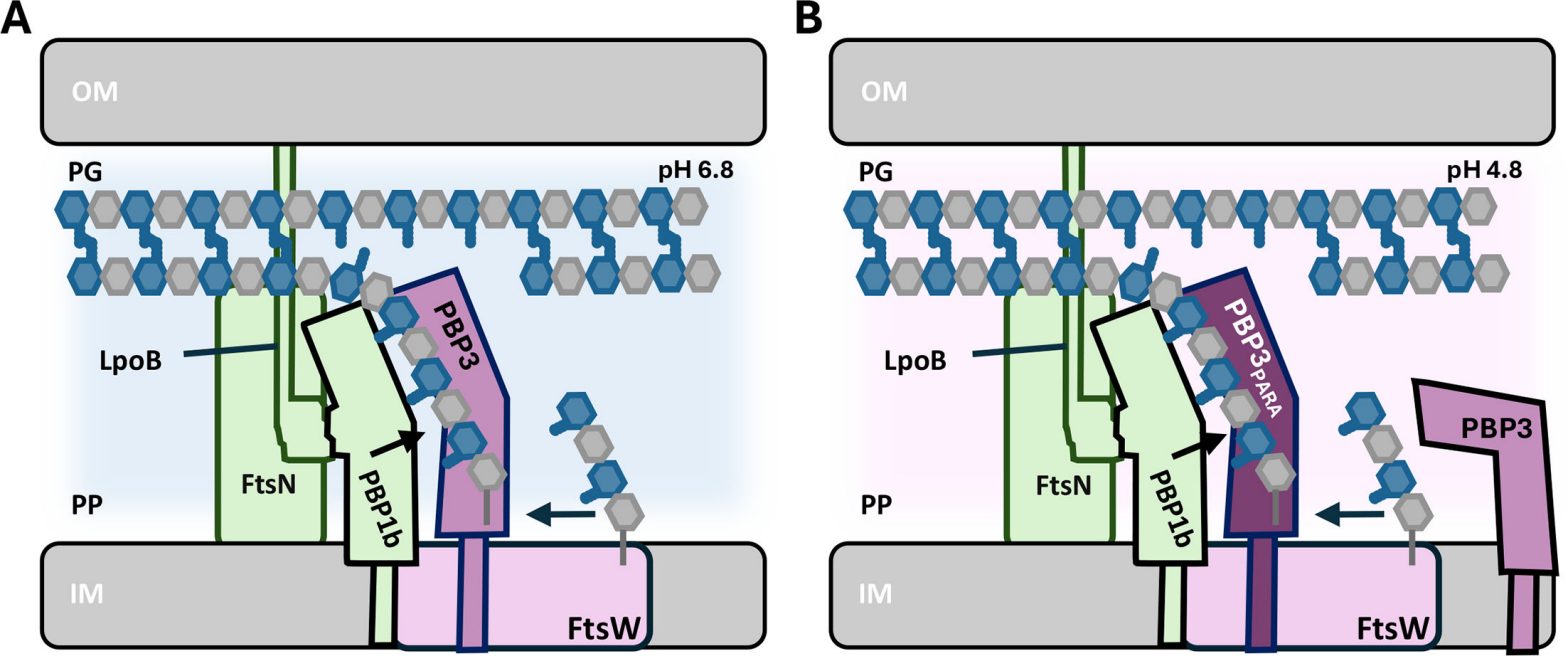
Model for divisome complex formation across pH conditions in *K. pneumoniae*. (**A**) At neutral pH, the canonical PBP3, in conjunction with FtsW and PBP1b, is responsible for septal PG synthesis. (**B**) During growth at low pH, *K. pneumoniae* expresses PBP3_PARA_ (encoded by *ftsI2*). Acidic environments favor associations between PBP3_PARA_, PBP1b, and FtsW, displacing the canonical PBP3 from the divisome. In the absence of interaction with FtsW, PBP3 assumes an inactive conformation, occluding its active site from interactions with beta-lactams.

Identification of paralogous class B PBPs has thus far only been described in pathogens (28–30, 48), but the role of these paralogous cell wall synthesis enzymes in pathogen physiology remains largely uncharacterized. In *Salmonella,* the acid-responsiveness of class B PBP paralogs raised speculation that they could be critical to support bacterial survival during intracellular growth within acidified host immune cells (29, 30, 49). Loss of PBP3_SAL_ did not impair replication within macrophages or attenuate virulence in a mouse infection model, presumably because the canonical PBP3 retains activity across all pH environments (29). Why *K. pneumoniae*’s canonical PBP3 is unable to fully support division in acidic conditions is unclear. The *K. pneumoniae* PBP3 shares 94% and 95% sequence identity to the canonical PBP3 in *E. coli* and *Salmonella,* respectively, both of which function at low pH. One potential explanation is that at low pH, *K. pneumoniae’s* canonical PBP3 is unable to optimally interact with key division partners, FtsW and PBP1b, thereby locking PBP3 in a closed conformation that impairs its transpeptidase activity (50) (Fig. 6). Additional work is required to fully elucidate the mechanisms underlying *K. pneumoniae* cell division, but our current results support a model in which the canonical PBP3 functions optimally at neutral pH while the paralogous PBP3_PARA_ functions predominately at acidic pH, with both working together to ensure that division remains robust across pH conditions.

In *Klebsiella pneumoniae,* our data suggest that class B PBP paralogs are part of a general strategy of functional redundancy to preserve critical enzymatic activity across a wide range of environmental conditions. The class B PBP paralogs modulate that resistance in part, suggesting that these pathogen-specific proteins may provide novel therapeutic targets. Targeting conditionally important, pathogen-specific proteins would be an attractive option to treat disease while sparing important commensal populations from unwanted killing by antibiotics.

We propose that future screens for new antimicrobial targets factor environmental pH as a variable to identify novel compounds that can bind to acid-responsive targets such as the class B PBP paralogs or to targets whose activity becomes more critical under low pH environments, such as PBP1b. Altogether, our results highlight the need to evaluate pathogen physiology in diverse environments beyond the standard laboratory conditions.

## MATERIALS AND METHODS

### Bacterial strains, growth conditions, and antibiotics

Bacterial strains and plasmids used in this study are listed in Tables S5 and S6. Strains were routinely cultured in lysogeny broth (LB; 1% Tryptone, 0.5% yeast extract, and 1% NaCl) in large glass tubes at 37°C with 200 RPM aeration. The pH of LB was adjusted with 1:10 MMT buffer (1:2:2 molar ratio of D/L-malic acid, MES, and Tris base). Artificial urine (AU) was prepared as described (19), and cells were cultured in either base AU or AU supplemented with all twenty amino acids at a final concentration consistent with what is used in EZ rich defined medium (https:www.genome.wisc.edu/resources/protocols/ezmedium.htm). The pH of AU or AU+AA was adjusted with MMT buffer immediately prior to use. For ease and clarity, AU media and amino acid compositions are listed in Table S7.

Where indicated, cultures were supplemented with kanamycin (50 µg/mL), spectinomycin (100 µg/mL), chloramphenicol (50 µg/mL), or IPTG (0.1 mM). The pRDC3 plasmid (p15A ori, SpecR, Plac) was a gift from Fabrizio Arigoni. Plasmid pSDB8 was generated from pRDC3 using the IVA cloning method to insert *ftsI2* under control of the Plac promoter (51). Primers were acquired from Integrated DNA Technologies (Coralville, IA), and sequences used are listed in Table S8. Chemically competent *E. coli* TOP10 (Invitrogen) were used to assemble vectors via IVA. Plasmid sequence was confirmed via whole plasmid sequencing (Plasmidsaurus) prior to transformation into *K. pneumoniae* strains.

For growth studies, cultures were started in LB supplemented with MMT buffer at the indicated pH and grown in large glass tubes at 37°C with 200 RPM aeration. Cultures were harvested once they reached mid-exponential phase (OD_600_ = 0.2–0.6), concentrated to an OD_600_ = 1.0, and serially diluted 10-fold. A volume of 1.5 µL of the 10^−5^ dilution was used to inoculate wells of sterile 96-well plates containing 150 µL of LB + MMT at the indicated pH. The plate was covered with a Breathe-Easy strip (Diversified Biosciences), and growth was monitored in a BioTek Synergy H1 plate reader with the following settings: 37°C, continuous double orbital shaking at 282 cycles per minute (cpm), with OD_600_ readings taken every 10 min.

The following antibiotics were used for antibiotic susceptibility screenings. Ampicillin sodium salt, aztreonam, cephalexin hydrate, cefsulodin sodium salt hydrate, meropenem trihydrate, and piperacillin sodium salt were obtained from Sigma Aldrich (St. Louis, MO). Carbenicillin sodium salt was from EMD Millipore (now Millipore Sigma, Germany). Doripenem was obtained from Cayman Chemicals (Ann Arbor, MI).

#### Genetic manipulation

Gene disruptions were carried out in the *K. pneumoniae* TOP52 background using a Lambda Red vector modified to carry a Spectinomycin resistance cassette as described previously (52, 53). Briefly, DreamTaq Polymerase (Thermo Scientific) was used to PCR amplify the kanamycin resistance cassette from the template plasmid pKD4 with primers containing 40–60 bp of homology to the chromosomal targets. Primers were designed to leave the start and stop codons of the targeted gene intact. In cases where two gene sequences overlapped, primers were designed to avoid disruption to any downstream genes. Linear PCR products were purified using a Purelink PCR purification kit (Invitrogen), and 2,000–3,000 ng of purified linear fragments were electroporated into *K. pneumoniae* cells grown in the presence of 0.7% L-arabinose to induce expression of the Lambda Red recombinase system from the pKD46S plasmid. Electroporated cells were recovered in SOC (Lloyd’s recipe—2% Tryptone, 0.5% yeast extract, 10 mM NaCl, 2.5 mM KCl, 10 mM MgSO_4_, 20 mM glucose) for 4–8 h at 30°C. Deletion mutants were selected by plating transformation culture onto LB agar plates containing kanamycin (50 µg/mL) and incubating at 37°C. Mutants were confirmed by colony PCR and sequencing as necessary. For marker-less deletions, cells were transformed with pCP20 at 30°C in LB + chloramphenicol (50 µg/mL) to express the FLP for excision of the FRT-flanked kanamycin cassette. Transformants were struck onto LB with no antibiotics and incubated overnight at 42°C to promote loss of pCP20. Spectinomycin, kanamycin, and chloramphenicol sensitivity was confirmed for strains prior to experimental use.

#### RNA sequencing

*K. pneumoniae* TOP52 or *E. coli* MG1655 was cultured overnight in LB buffered to either pH 4.8 or 6.8 with MMT buffer. Overnight cultures were back-diluted 1:100 into fresh media and cultured at 37°C with 200 RPM aeration until cultures reached the early exponential phase (OD_600_ = ~0.2). Approximately 9.6 × 10^8^ cells were transferred to 15 mL conical Falcon tubes and pelleted by centrifugation at 4°C (4,000 RPM for 10 min). The supernatant was discarded, cell pellets were resuspended in 350 uL of RNAwiz, and the RNA was extracted following the protocol for the Ribopure-Bacteria RNA extraction kit (Invitrogen). RNA was eluted in 50 µL of elution solution. Purified RNA was subjected to a DNase I treatment to remove any contaminating chromosomal DNA. RNA sequencing and differential gene expression analysis were performed by MiGS (now SeqCenter). Quality control and adapter trimming of reads were performed with bcl2fastq. Read mapping was performed with HISAT2, and read quantification was performed using Subread’s featureCounts. Read counts were normalized in R using edgeR’s Trimmed Mean of M values algorithm, and subsequent values were converted to counts per million (CPM). Differential expression analysis was performed using edgeR’s Quasi-Linear F-Test (qlfTest) functionality against treatment groups, and DEGs were considered as those with |log_2_FC| >1 and *P* value <0.05. Three biological replicates were submitted per pH condition.

#### Antibiotic susceptibility testing

Cells were cultured from a single colony in LB at the indicated pH to the exponential phase (OD_600_ = ~0.2–0.6) at 37°C with 200 RPM aeration. Harvested cells were concentrated to an OD_600_ = 1 and serially diluted 10-fold. A volume of 1.5 µL of the 10^−2^ dilution was used to seed wells containing 150 µL of LB broth + MMT (matching pH) in sterile 96-well microtiter plates containing a range of twofold dilutions of beta-lactam antibiotics. Plates were sealed with a Breathe-Easy strip (Diversified Biotech) and incubated for 20 h at 37°C with 200 RPM aeration. The MIC was determined by visual inspection and reflects the lowest drug concentration that completely inhibited bacterial growth.

For MICs in AU or AU + AA, overnight cultures were back-diluted 1:100 into fresh media and cultured at 37°C with 200 RPM aeration at the indicated pH and were harvested once cultures reached the exponential phase (OD_600_ = ~0.2–0.6).

Strains carrying expression vectors were grown overnight without inducer in LB at the desired pH and with appropriate antibiotics. Overnight cultures were back-diluted into fresh media with appropriate antibiotics and 0.1 mM IPTG for induction and incubated at 37°C with 200 RPM aeration until cells were harvested in the exponential phase (OD_600_ = ~0.2–0.6). MICs were performed in the presence of inducer (0.1 mM IPTG) and appropriate antibiotics for plasmid maintenance.

#### Terminal morphology assessment

At the end of 20 h for antibiotic susceptibility tests, cells were taken from wells following growth at either neutral or acidic pH with the indicated antibiotic concentrations. Five microliters of cells was spotted onto LB + 1% agarose and imaged by phase contrast microscopy.

#### Microscopy, cell size analysis, and time-lapse imaging

All phase contrast and fluorescence images were acquired on either a Nikon Ti-E inverted microscope or a Ti2-E inverted microscope (Nikon Instruments). The Ti-E is equipped with a 100× Plan Apo oil objective (N.A. = 1.45 Ph3 objective), X-Cite 120 LED light source (Lumen Dynamics), and an OrcaERG CCD camera (Hammamatsu Photonics, Bridgewater, NJ). Filter cubes were purchased from Chroma Technology Corporation. The Ti2-E inverted microscope is equipped with a 100× Plan Apo oil objective (N.A. = 1.45, Ph3 objective), Lumencor Sola LED light engine (Lumencore), and an orca-Fusion sCMOS camera (Hammamatsu Photonics). Filter cubes were purchased from Chroma Technology Corporation. Both microscopes have an enclosed temperature-controlled enclosure to pre-heat the objective to 37°C (OXO enclosure) prior to experiments. Nikon Elements software was used for image capture, and analysis was performed using the MicrobeJ plug-in in ImageJ.

For cell size analysis, cells were grown from a single colony in LB with MMT buffer at the indicated pH at 37°C and 200 RPM aeration to the exponential phase (OD_600_ = 0.2–0.4) and back-diluted to an OD_600_ = 0.005 into 7 mL of pre-warmed LB + MTT (same pH). Growth was monitored via optical density, and when cultures reached the early exponential phase (OD_600_ = 0.1–0.2), cells were harvested. Five microliters of cells was spotted onto LB + MTT (same pH) + 1% agarose pads and imaged by phase contrast microscopy. Cell morphology and size dimensions were determined using the MicrobeJ (54) plug-in for ImageJ.

For p*ftsI2* cell size complementation studies, un-induced overnight cultures of strains carrying either pRDC3 (empty vector [EV]) or pSDB8 were back-diluted 1:500 into 3 mL of fresh LB buffered to either pH 4.8 or pH 6.8 containing spectinomycin (100 µg/mL). Cultures were grown at 37°C with 200 RPM aeration for 1.5 h. Expression of *ftsI2* was induced with the addition of 0.1 mM IPTG, and cultures were allowed to continue growing until the early exponential phase (OD_600_ = 0.1–0.3) was reached (~40–90 additional minutes). Cultures were back-diluted into fresh buffered media containing spectinomycin (100 µg/mL) and 0.1 mM IPTG to an OD_600_ ≅ 0.005. Growth was monitored every 30 min, and cells were harvested when cultures reached the early exponential phase (OD_600_ = 0.1–0.3). Cells were spotted onto media pads (LB + MMT [pH 4.8 or 6.8] + spectinomycin [100 µg/mL] + 0.1 mM IPTG + 1% agarose) and imaged live with phase microscopy. Due to the increased acid sensitivity of the Δ*ompR* strain, 1.5 µM propidium iodide was added to the agarose pads for p*ftsI2* complementation studies. Cells that stained with propidium iodide were eliminated from the cell size quantification.

For time-lapse imaging experiments, cultures were grown from a single colony in LB with MMT buffer at the indicated pH at 37°C and 200 RPM aeration to the exponential phase (OD_600_ = 0.1–0.4). Five microliters of cells was spotted onto LB + MTT (same pH) + 1% agarose pads that contain antibiotics at indicated concentrations and 1.5 µM propidium iodide (PI) for monitoring loss of membrane integrity and covered with a #1.5 coverslip. Phase images were obtained every 2 min, and fluorescent images were collected every 10 min (Chroma AT560/40X/Chroma AT600 DC/Chroma at635/60nm with 100 mS exposure/10% intensity). For each experiment, a total of 2–5 different XY coordinates were monitored throughout the duration of the experiment. Quantification of PI^+^ cells was done using the MicrobeJ (54) plug-in for ImageJ. Briefly, the number of cells in the phase image and the number of corresponding fluorescent cells were quantified. Data were reported as either percentage of PI^+^ cells (number of PI^+^ cells/total number of cells × 100) or percent alive (number of cells − PI^+^ cells/total number of cells × 100).

### Statistical analysis

Statistical analyses were either performed in Graphpad Prism or R.

## Data Availability

All raw and processed data required to evaluate the conclusions of this study are included in the text or supplemental data. The RNA sequencing data for gene expression studies at acidic and neutral pH have been deposited in the NCBI GEO repository: GSE291667.

## SUPPLEMENTAL MATERIAL

Supplemental material mbio.00092-26-s0001.docx Tables S5 and S6, Fig. S1-S9, and captions for supplemental videos.

Supplemental Tables mbio.00092-26-s0002.xlsx Tables S1 and S2.

Table S3 mbio.00092-26-s0003.xlsx Differentially regulated genes during growth in LB at pH 4.8 versus pH 6.8 in both *E. coli* MG1655 and *K. pneumoniae*.

Table S4 mbio.00092-26-s0004.xlsx Beta-lactam MICs for *K. pneumoniae* TOP52 and mutants in LB at pH 4.8 and 6.8.

Table S7 mbio.00092-26-s0005.xlsx Composition of artificial urine and amino acid stocks used to make base AU and AU+AA media.

Table S8 mbio.00092-26-s0006.xlsx Primer sequences used in this study.

Video S1 mbio.00092-26-s0007.avi Phase-contrast time lapse that corresponds to micrographs in the top panel of Fig. S5.

Video S2 mbio.00092-26-s0008.avi Fluorescent time-lapse data that correspond to micrographs in the top panel of Fig. S5.

Video S3 mbio.00092-26-s0009.avi Phase-contrast time lapse that corresponds to micrographs in the bottom panel of Fig. S5.

Video S4 mbio.00092-26-s0010.avi Corresponds to micrographs in the bottom panel of Fig. S5.

ASM does not own the copyrights to Supplemental Material that may be linked to, or accessed through, an article. The authors have granted ASM a non-exclusive, world-wide license to publish the Supplemental Material files. Please contact the corresponding author directly for reuse.
