# Supplementary material for: Acid-dependent beta-lactam resistance in Klebsiella pneumoniae is mediated by paralogous class B PBPs and the class A PBP, PBP1b
Source: mBio. 2026 May 11;17(6):e00092-26. doi: 10.1128/mbio.00092-26 (PMC13251458; doi:10.1128/mbio.00092-26)

**Supplemental table legends:**

**Table S1.** Beta-lactam MICS for *K. pneumoniae* TOP52 in LB across five pH values. Corresponds to heatmap data presented in Fig 1.

**Table S2.** Beta-lactam MICs for *K. pneumoniae* TOP52 in artificial urine (AU) and artificial urine supplemented with amino acids (AU+AA) at pH 4.8 and 6.8. Data corresponds to Fig S2.

**Table S3.** List of differentially regulated genes (DEG) during growth in LB at pH 4.8 versus pH 6.8 in both *E. coli* MG1655 and *K. pneumoniae.*

**Table S4.** Beta-lactam MICs for *K. pneumoniae* TOP52 and mutants in LB at pH 4.8 and 6.8. Data corresponds to Fig 4, 5, and Fig S7.

**Table S5.** Strains used in this study.

**Table S6.** Plasmids used in this study.

**Table S7.** Composition of Artificial urine and amino acid stocks used to make base AU and AU+AA media.

**Table S8.** Primer sequences used in this study.

**Table S5.** Strains used in this study.

| Strain ID | Genotype | Source |
| --- | --- | --- |
| PAL4422 | *Klebsiella pneumoniae* TOP52 | ^1,2^ |
| PAL4649 | NTUH-K2044 | ^3^ |
| PAL4615 | ATCC 43816 | ^2^ |
| SDB583 | *K. pneumoniae* TOP52 Δ*bla*::kan | This study |
| SDB595 | *K. pneumoniae* TOP52 Δ*mrcA*::kan | This study |
| SDB597 | *K. pneumoniae* TOP52 Δ*mrcB*::kan | This study |
| SDB624 | *K. pneumoniae* TOP52 Δ*pbpC*::kan | This study |
| SDB676 | *K. pneumoniae* TOP52 Δ*mrdA2*::kan | This study |
| SDB689 | *K. pneumoniae* TOP52 Δ*ftsI2*::kan | This study |
| SDB588 | *K. pneumoniae* TOP52 Δ*dacA*::kan | This study |
| SDB591 | *K. pneumoniae* TOP52 Δ*dacD*::kan | This study |
| SDB700 | *K. pneumoniae* TOP52 Δ*ftsI2*::frt Δ*mrdA2*::kan | This study |
| SDB767 | *K. pneumoniae* TOP52 *ΔlpoB*::kan | This study |
| SDB774 | *K. pneumoniae* TOP52 *ΔompR*::kan | This study |
| SDB737 | PAL4422/pRDC3 | This study |
| SDB739 | SDB689/pRDC3 | This study |
| SDB745 | PAL4422/pSDB8 | This study |
| SDB747 | SDB689/pSDB8 | This study |
| SDB778 | SDB774/pRDC3 | This study |
| SDB780 | SDB774/pSDB8 | This study |
| PAL2036 | *Escherichia coli* MG1655 | ^4^ |

**Table S6.** Plasmids used in this study.

| **Plasmid ID** | **Genotype/Annotation** | **Source** |
| --- | --- | --- |
| pRDC3 | Empty vector, Plac, SpecR | Gift from Fabrizio Arigoni |
| pSDB8 | pRDC3, *Plac::ftsI2* | This study |
| pKD46S | Lambda Red Recombinase, SpecR | ^5,6^ |
| pKD4 | Source of Kanamycin Resistance cassette | ^6^ |
| pCP20 | Source of FLP | ^6^ |

**References:** 1. Ko, D. C. *et al.* Whole-Genome Sequencing of *Klebsiella pneumoniae* Isolates to Track Strain Progression in a Single Patient With Recurrent Urinary Tract Infection. *Frontiers in Cellular and Infection Microbiology*  **9**, 14 (2019).

2. Rosen, D. A. *et al.* *Klebsiella pneumoniae* FimK Promotes Virulence in Murine Pneumonia. *The Journal of Infectious Diseases* **213**, 649–658 (2016).

3. Chou, H.-C. *et al.* Isolation of a Chromosomal Region of *Klebsiella pneumoniae* Associated with Allantoin Metabolism and Liver Infection. *Infection and Immunity* **72**, 3783–3792 (2004).

4. Guyer, M. S., Reed, R. R., Steitz, J. A. & Low, K. B. Identification of a Sex-factor-affinity Site in *E.* *coli* as γδ. *Cold Spring Harb Symp Quant Biol* **45**, 135–140 (1981).

5. Bachman, M. A. *et al.* Genome-wide identification of *Klebsiella pneumoniae* fitness genes during lung infection. *mBio* **6**, (2015).

6. Datsenko, K. A. & Wanner, B. L. One-step inactivation of chromosomal genes in *Escherichia coli* K-12 using PCR products. *Proceedings of the National Academy of Sciences* **97**, 6640–6645 (2000).

**Supplemental figure legends**

**Fig S1. Quantification of cell shape changes following beta-lactam treatment at low and neutral pH.** (A) Quantification of cell roundness following Meropenem exposure at low (n=4,178) and neutral pH (n=2,697). Values of 0 correspond to rods and values of 1 correspond to complete spheres. Line represents median values. (B) Quantification of cell length following Cephalexin exposure at low (n=1,020) and neutral pH (n=844). Line represents mean values. Statistical significance was determined by a Mann-Whitney U test with asterisks denoting significance as follows : **** = p <0.0001. NS = Not significant.

**Fig S2. *K. pneumoniae* exhibits acid-dependent beta-lactam resistance in a variety of nutrient environments**. Heatmap displaying the fold change in MIC for the indicated beta-lactams at pH 4.8 compared to pH 6.8 in indicated media conditions (LB = Lysogeny Broth, AU + AA = Artificial Urine supplemented with amino acids, and AU = Artificial Urine). Higher values (magenta) indicate increased MIC at low pH, while values of 1 indicate the MIC is the same at both pH conditions. AMP = Ampicillin, DOR = Doripenem, MER = Meropenem, and CEX = Cephalexin. The exact fold change for CEX could not be determined in AU ± AA as there was growth in all concentrations tested – see also Supplemental table 4.

**Fig S3. Sequence and domain conservation between canonical and paralogous class B PBP proteins.** (A & B) Protein sequence conservation between canonical and paralogous copies of PBP2 (B) and PBP3 (A). Purple bars indicate residues that are critical for catalysis. (C & D) Domain conservation between canonical and paralogous copies of PBP2 (C) and PBP3 (D). Domains are denoted as follows: Transmembrane (TM), Lipoprotein lipid attachment site (LP, PS51257) PBP-dimer (Pfam: PF03717), Transpeptidase domain (Pfam: PF00905). Location of motifs that are critical for enzyme catalysis are highlighted in purple.

**Fig S4. Distribution of cell lengths in Δ*ftsI2* populations during growth at low pH.** (A) Histograms showing the distributions of *K. pneumoniae ΔftsI2* cells grown at pH 4.8. A minimum of 50 cells were analyzed for each biological replicate. Bin lengths indicate the maximum length (i.e. a bin length of 5 includes all cells with a length of 1-5 µM). (B) Micrographs of *K. pneumoniae ΔftsI2* cells grown at pH 4.8. Agarose pads are comprised of LB adjusted to pH 4.8 with MMT buffer + 1% agarose. Scale bar = 10 µM.

**Fig S5. Acid-grown Δ*ftsI2* filamentous cells resume division upon transition from acidic to neutral pH. (**A-B) Micrographs of Δ*ftsI2* cultured in LB+MMT, pH 4.8 spotted onto (A) LB+MMT, pH 4.8 + 1% agarose pads with 1.5 µM propidium iodide or (B) LB+MMT, pH 6.8 + 1% agarose pads with 1.5 µM propidium iodide

**Fig S6. Loss of *mrcB* does not significantly alter growth at low pH in *K. pneumoniae.*** Growth of *K. pneumoniae* WT (circles) or Δ*mrcB* (open squares) at pH 4.8 (A), pH 5.5 (B), or pH 6.8 (C). Line represents mean values of n=4 biological replicates with error bars denoting standard deviation.

**Fig S7. Loss of PBP1b or its outer membrane activator LpoB impairs acid-dependent Beta-lactam resistance.** (A) Fold change (FC) in Doripenem (DOR) MICs at pH 4.8 relative to pH 6.8 shown for class A PBP mutants with loss of PBP1a (Δ*mrcA*) or PBP1b (ΔmrcB). (B) Fold change (FC) in MIC of indicated beta-lactams at pH 4.8 compared to pH 6.8 for a Δ*lpoB* mutant. AMP = Ampicillin, CEF = Cefsulodin, MER = Meropenem, DOR = Doripenem, and CEX = Cephalexin. All data are graphed as median with range.

**Fig S8. Complementation of *ftsI2* in Δ*ftsI2* mutant restores WT-level response to beta-lactams at low pH.** (A-B) MICs for (A) Ampicillin (AMP) or (B) Piperacillin (PIP) at pH 4.8 for indicated strains carrying either an empty vector control (EV) or an IPTG-inducible copy of *ftsI2* (p*ftsI2*). Data are graphed as median values with range.

**Fig S9. Induction of p*ftsI2* in WT or Δ*ftsI2* mutant does not alter resistance to Cephalexin at neutral pH.**(A) Cephalexin MICs at neutral pH for indicated strains carrying either an empty vector control (EV) or an IPTG-inducible copy of *ftsI2* (p*ftsI2*). Data are graphed as median values with range.

**Supplemental movies 1 and 2.** Corresponds to micrographs in the top panel of Fig S5. *K. pneumoniae* Δ*ftsI2* cells were cultured in LB+MMT, pH 4.8 until early exponential phase (OD_600_ = 0.1-0.2) and then 5 µL were spotted onto LB + 1% agarose pads buffered to pH 4.8 containing 1.5 µM propidium iodide to monitor membrane integrity. Scale bar = 10 µm. Movie 1 corresponds to the phase contrast images taken every 2 minutes, and movie 2 is the corresponding fluorescent images taken every 10 minutes.

**Supplemental movies 3 & 4.** Corresponds to micrographs in bottom panel of Fig S5. *K. pneumoniae* Δ*ftsI2* cells were cultured in LB+MMT, pH 4.8 until early exponential phase (OD_600_ = 0.1-0.2) and then 5 µL were spotted onto LB + 1% agarose pads buffered to pH 6.8 containing 1.5 µM propidium iodide to monitor membrane integrity. Scale bar = 10 µm. Movie 1 corresponds to the phase contrast images taken every 2 minutes, and movie 2 is the corresponding fluorescent images taken every 10 minutes.

**Supplemental Figures**

Fig S1


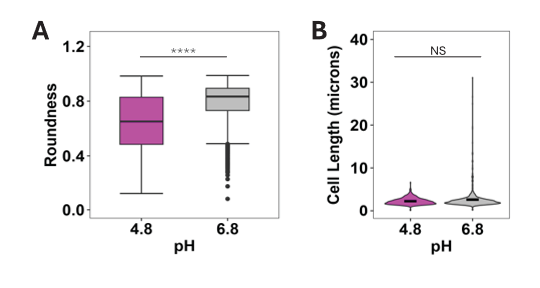


Fig S2


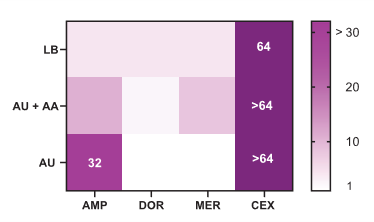


Fig S3


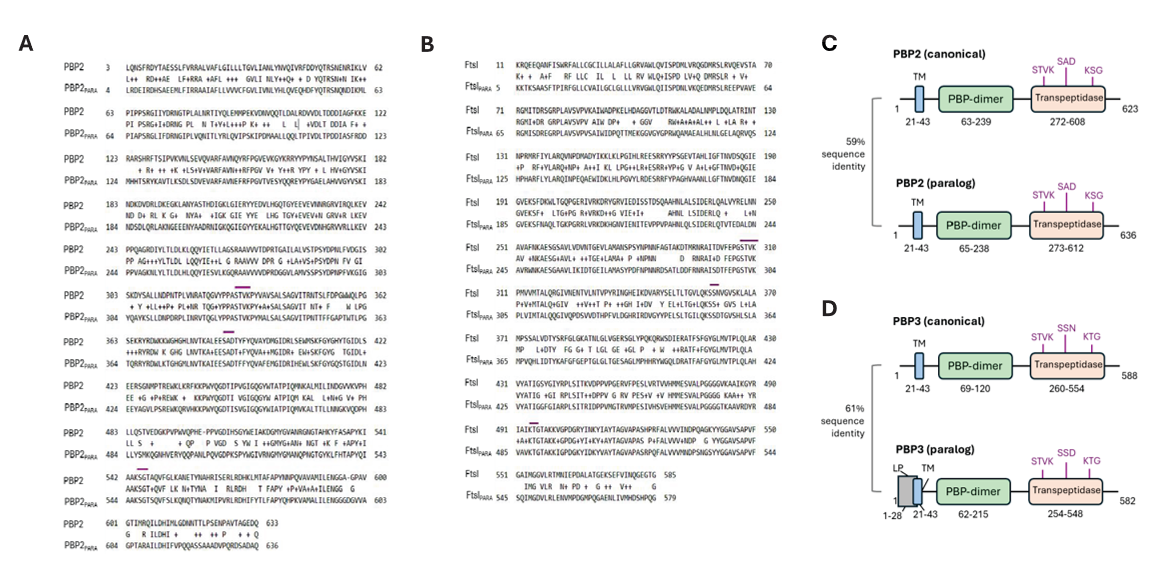


Fig S4


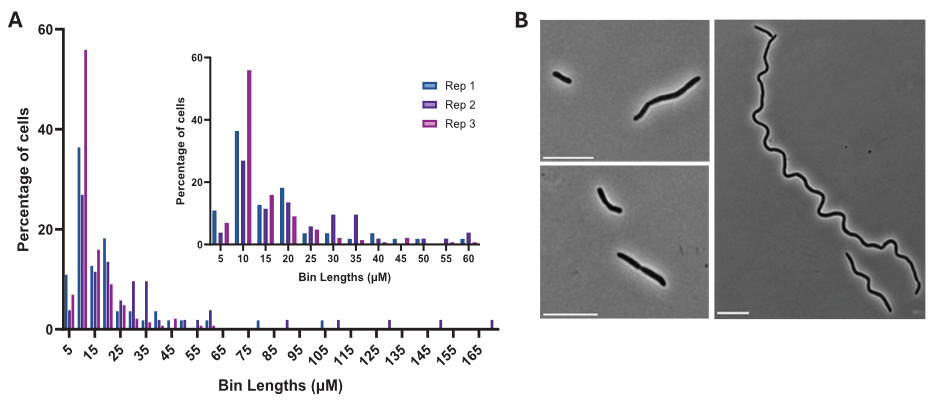


Fig S5


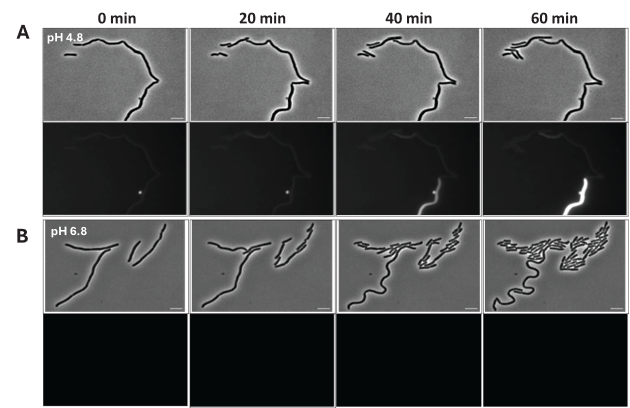


Fig S6


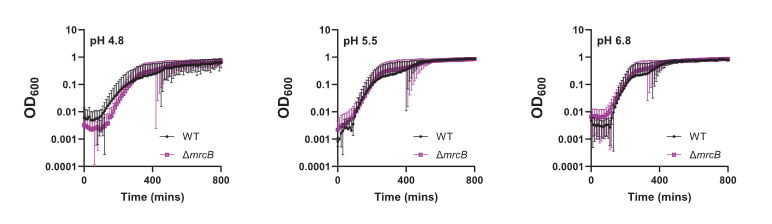


Fig S7


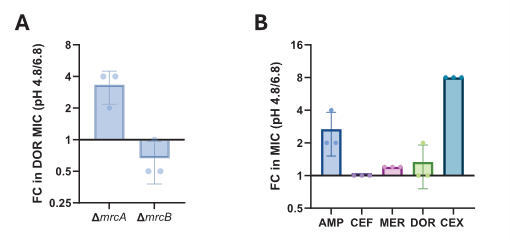


Fig S8


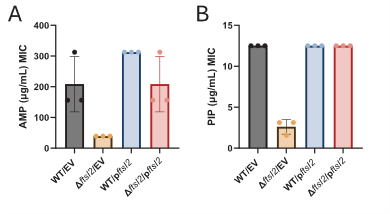


Fig S9


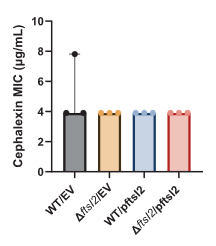

Supplement: Supplemental material — Tables S5 and S6, Fig. S1-S9, and captions for supplemental videos. [file mbio.00092-26-s0001.docx]
